# The relation between dialysis-requiring acute kidney injury and recovery from end-stage renal disease: a national study

**DOI:** 10.1186/s12882-019-1483-y

**Published:** 2019-09-02

**Authors:** Zijin Chen, Benjamin J. Lee, Charles E. McCulloch, Nilka Ríos Burrows, Michael Heung, Raymond K. Hsu, Meda E. Pavkov, Neil R. Powe, Rajiv Saran, Vahakn Shahinian, Chi-yuan Hsu, for the Centers for Disease Control and Prevention Chronic Kidney Disease Surveillance Team

**Affiliations:** 1 Division of Nephrology, Ruijin Hospital Affiliated to Shanghai Jiaotong University School of Medicine, Shanghai, China; 2 Division of Nephrology, Department of Medicine, University of California, San Francisco, 533 Parnassus Ave, U404, Box 0532, San Francisco, CA 94143-0532 USA; 3 Houston Kidney Consultants, Houston, TX USA; 4 Houston Methodist Institute for Academic Medicine, Houston, TX USA; 5 Department of Epidemiology and Biostatistics, University of California, San Francisco, CA USA; 6 Division of Diabetes Translation, Centers for Disease Control and Prevention, Atlanta, GA USA; 7 Division of Nephrology, Department of Medicine, University of Michigan, Ann Arbor, MI USA; 8 UCSF Center for Vulnerable Populations, Department of Medicine, University of California, San Francisco, CA USA; 9 Department of Medicine, San Francisco General Hospital, San Francisco, CA USA; 10 Kidney Epidemiology and Cost Center, University of Michigan, Ann Arbor, MI USA

**Keywords:** AKI-D, ESRD, Renal recovery

## Abstract

**Background:**

Approximately 4–6% of incident end stage renal disease (ESRD) patients in the U.S. recover enough kidney function to discontinue dialysis but there is considerable geographic variation. We undertook this study to investigate whether state-level variations in renal recovery among incident ESRD patients correlated with state-level variations in incidence of acute kidney injury requiring dialysis (AKI-D).

**Methods:**

We conducted a national cross-sectional ecological study at the state-level using data from State Inpatient Databases and U.S. Renal Data System. All hospital admissions and all ESRD patients in 18 US states (AZ, AR, CA, FL, IA, KY, MA, MD, MI, NJ, NM, NY, NV, OR, RI, SC, VT, and WA) were included. Correlation between AKI-D incidence and rate of renal recovery across states was determined using Pearson’s r (overall and in subgroups). We also calculated partial correlations adjusted for sex and age.

**Results:**

AKI-D incidence ranged from 99.0 per million population (pmp) in Vermont to 490.4 pmp in Nevada. Rate of renal recovery among incident ESRD patients ranged from 8.8 pmp in Massachusetts to 29.3 pmp in Florida. A positive correlation between AKI-D incidence and rate of renal recovery among incident ESRD patients at state level was found overall (unadjusted *r* = 0.67; *p* = 0.002) and in age, sex, and race subgroups. The overall correlation persisted after adjusting for age (adjusted *r* = 0.62; *p* < 0.001) and sex (adjusted *r* = 0.65; *p* < 0.001).

**Conclusion:**

Our findings suggest that AKI-D incidence is an important driver of renal recovery rates among incident ESRD patients.

**Electronic supplementary material:**

The online version of this article (10.1186/s12882-019-1483-y) contains supplementary material, which is available to authorized users.

## Background

Recent studies suggest that the rate of renal recovery among patients on maintenance dialysis has increased over the last two decades such that 4–6% of contemporary patients registered with incident end stage renal disease (ESRD) in the United States become dialysis independent within 1 year [1, 2]. This somewhat surprising observation may reflect an increasing number of patients entering the ESRD program after acute kidney injury (AKI) requiring dialysis (AKI-D) who subsequently recovered kidney function over a period of months.

Prior research also reported geographic variation in renal recovery rates among incident ESRD patients in the United States [2] but the reasons behind this observation are not well understood. Therefore, we undertook this study to investigate whether state-level variations in AKI-D correlated with state-level variations in renal recovery among incident ESRD patients.

## Methods

### Study design

We conducted a national, cross-sectional, ecologic study of AKI-D and renal recovery among incident ESRD patients in the United States.

### Determination of number of AKI-D hospitalizations at state level

We used State Inpatient Databases (SID) to determine the number of AKI-D hospitalizations [3]. SID are part of the family of databases and software tools developed for the Healthcare Cost and Utilization Project (HCUP), developed through a U.S. Federal-State-Industry partnership. The SID contain the universe of the inpatient discharge abstracts in participating States, translated into a uniform format to facilitate multi-State comparisons and analyses. The SID contain a core set of clinical and nonclinical information on all patients, regardless of insurance satus [3–5].

Although there were 30 SID databases available for 2011, we only had financial resources to purchase 25 of them. We selected a convenience sample of 25 of the larger states by geographic region (which captured 90% of discharges): Arizona (AZ), Arkansas (AR), California (CA), Colorado (CO), Florida (FL), Iowa (IA), Kentucky (KY), Massachusetts (MA), Maryland (MD), Maine (ME), Michigan (MI), Mississippi (MS), North Carolina (NC), Nebraska (NE), New Jersey (NJ), New Mexico (NM), New York (NY), Nevada (NV), Oregon (OR), Rhode Island (RI), South Carolina (SC), Utah (UT), Vermont (VT), Washington (WA), and West Virginia (WV)(Additional file 1: Figure S1). The different state databases varied in capturing whether a diagnosis was present on admission or not and in indicating whether an admission represented a re-admission to the hospital for the same patient in a given calendar year.

We defined AKI-D as requiring both a diagnostic code for acute renal failure (International Classification of Diseases, Ninth Revision, Clinical Modification [ICD-9] codes 584.5, 584.6, 584.7, 584.8, or 584.9) and a procedure code for dialysis (39.95, V45.11, V45.12, V56.0, or V56.2), along with the absence of procedure codes for arteriovenous fistula creation or revision (39.27, 39.42, 39.43, or 39.93) [6–8]. This algorithm has been shown to be sensitive and specific, producing high positive and negative predictive values (all ≥90%) [6–8].

To address potential ascertainment bias arising from the fact that states reported different numbers of diagnostic codes (range 9–60) and different numbers of procedure codes (range 6–30) for each individual in these databases, we first analyzed the number of actual diagnostic codes and procedure codes for each hospitalization. Of the 25 SID databases, 19 (AZ, AR, CA, CO, FL, IA, KY, MD, MI, NV, NJ, NM, NY, NC, OR, RI, VT, WA, and WV) reported > 15 diagnostic codes. In none of these 19 states did more than a quarter of the hospitalizations have > 15 diagnostic codes (range 4.1–24.5%). Of the 25 SID databases, 19 states (AZ, AR, CA, CO, FL, IA, KY, MD, MA, MI, NV, NJ, NY, NC, OR, RI, SC, VT, and WA) reported > 6 procedure codes. In none of these 19 states did more than a tenth of the hospitalizations have > 6 procedure codes (range 1.6–6.1%).

Thus in our primary analysis, we excluded the 2 states with fewer than 15 diagnostic codes (ME, NE) (Additional file 1: Figure S1). All states had at least 6 procedure codes. For the remaining states, we only analyzed the first 15 diagnostic codes and first 6 procedure codes listed for each hospitalization (i.e. for states whose database contained additional information, we ignored the diagnostic codes in position 16 and above and we ignored procedure codes in position 7 and above).

We did not count as AKI-D hospitalizations those hospitalizations with a diagnostic code for ESRD present on admission (585.6). Thus, in our primary analysis, we additionally excluded 5 states (CO, MS, NC, UT, WV) whose SID did not specify whether a diagnosis of ESRD was present on admission or not) (Additional file 1: Figure S1). (We did include AKI-D hospitalizations with diagnosis of ESRD only on discharge but not on admission.)

Therefore, our primary analysis was based on 18 states (AZ, AR, CA, FL, IA, KY, MA, MD, MI, NJ, NM, NY, NV, OR, RI, SC, VT, WA) (Additional file 1: Figure S1). In 2011, these 18 states accounted for 50% of the country’s incident ESRD cases [9].

In sensitivity analyses, we used data from all 25 states we had SID data on but to make ascertainment more uniform, we only analyzed up to 9 diagnostic codes and up to 6 procedure codes for each state (all states reported at least these numbers of codes). In this sensitivity analysis, we used the same AKI-D definition as above, but we excluded all hospitalizations containing a diagnostic code for ESRD (585.6) regardless of whether it was present on admission or not (since for CO, MS, NC, UT, and WV, we could not tell if a given diagnosis was present on admission or not).

### Determination of incidence of AKI-D per state

To calculate AKI-D incidence, we used as denominator state populations according to US Census Bureau [10, 11].

We considered AKI-D hospitalization per year as being equal to number of patients who had AKI-D per year [12]. We based this on the fact that in the 8 states in which readmission can be identified (using the ‘VisitLink’ variable in the AR, CA, FL, IA, MA, NM, NY, VT, and WA databases), only 0.0–8.7% of patients had more than one AKI-D hospitalization*.*

### Determination of number of renal recovery cases among incident ESRD patients

The US Renal Data System (USRDS) is a national registry that includes virtually all patients with treated ESRD in the United States [13]. We defined renal recovery among incident ESRD patients as a reported treatment modality of “recovered function” ≥ 90 days in the absence of renal transplant or death, within 1 year of dialysis initiation [1].

In additional sensitivity analyses, we used a broader definition of recovery, grouping the “recovered function,” “discontinued dialysis,” and “lost to follow-up” modalities together as time in a recovered state. Patients who remained alive in this recovered state for ≥90 days without a renal transplant were counted as recovered [1]. Finally, we also examined only recovery within 6 months and not 12 months after initiation of dialysis.

### Determination of number of nephrologists per state

To explore whether the absence of renal recovery among incident ESRD patients might be related to the number of nephrologists (i.e. supplier-induced demand) and thus might be a state-level confounder, we also examined if the number of nephrologists in each state in 2011 was associated with renal recovery. We determined the number of nephrologists using the American Medical Association (AMA) Physician Professional Data (PPD) Statistical Research file [14, 15] which includes current and historical data for more than 1.4 million physicians, residents, and medical students in the United States and their mailing addresses [14, 15]. Physicians known to be retired, deceased, or in a training program through the end of 2011 were excluded.

### Statistical approach

The AKI-D incidence and rate of renal recovery among incident ESRD patients were both expressed as per million population (pmp) per year for each state. We used Pearson correlations to analyze the association between AKI-D incidence and rate of renal recovery across states. We repeated our analyses in subgroups defined by sex, age (45–64, 65–74, ≥75 years). We did not show results for age 0–44 years old due to small number of outcomes (for example, 8 of the 18 states had fewer than 10 observed cases of renal recovery among incident ESRD patients of this age range).

We also did not show results stratified by race/ethnicity as 10 of the 18 states had fewer than 10 observed cases of renal recovery among incident, non-Hispanic black ESRD patients. We also used partial correlation to analyze AKI-D incidence and the rate of renal recovery across states adjusted by sex and age groups. (We did not adjust for race as there was no correlation between race and AKI-D incidence and no correlation between race and rate of renal recovery.)

Data were analyzed using SPSS version 23.0 (IBM SPSS, Chicago, IL). Results were independently confirmed using SAS version 9.4 (SAS Institute Inc., Cary, NC) or STATA version 14.1 (College Station, TX) by separate analysts.

## Results

In our primary analysis using 18 states in 2011, we identified 38,591 AKI-D hospitalizations and 2,746 incident ESRD patients with renal recovery (Table 1).

**Table 1:** Characteristics of dialysis-requiring acute kidney injury (AKI-D) hospitalizations and recovered end-stage renal disease (ESRD) patients across 18 states in 2011

	AKI-D	Recovered ESRD
N	38,591	2,746
Median age, yrs. (25, 75%)	66 (54, 77)^a^	64 (53, 73)
Male %	58.1	61.1
Race/ethnicity		
Non-Hispanic black, %	14.7	16.0
Non-Hispanic white, %	63.7	64.2
Hispanic, %	11.4	15.5
Asian, %	3.2	3.4
Missing, %	4.0	0.9
Primary cause of ESRD		
Diabetes Mellitus, %	/	24.4
Tubular necrosis, %	/	24.0
Multiple myeloma or light chain disease, %	/	2.8
Acute interstitial nephritis	/	2.1

*Yrs* years

^a^Excluding one state which only reported age categories

**Fig. 1:**
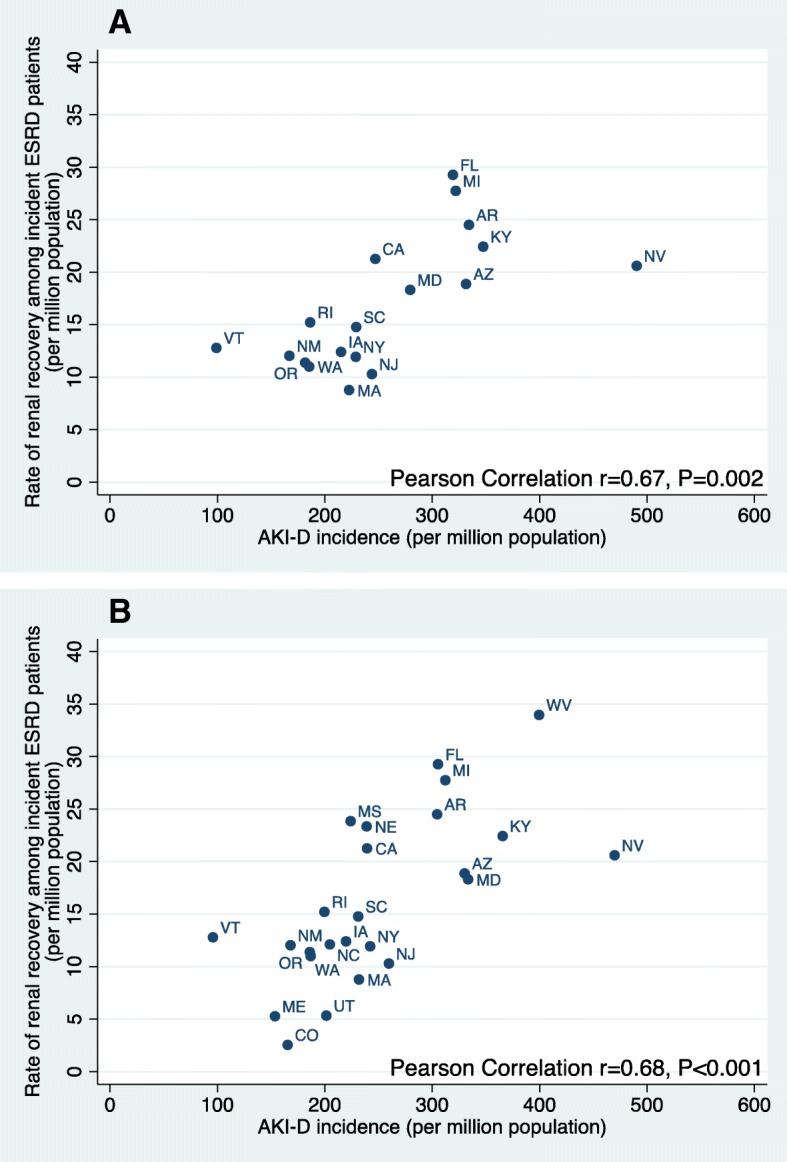
Dialysis-requiring acute kidney injury (AKI-D) incidence per million population vs. rate of renal recovery among incident end-stage renal disease (ESRD) patients per million population by state in 2011. **a** Primary analysis (18 states) (also see Additional files 1: Table S1), **b** Sensitivity analysis (25 states)

There was clear geographic variation among the states in both AKI-D incidence and rate of renal recovery among incident ESRD patients (Fig. 1; Additional file 1: Table S1). AKI-D incidence ranged from 99.0 pmp in Vermont to 490.4 pmp in Nevada. While the rate of renal recovery among incident ESRD patients ranged from 8.8 pmp in Massachusetts to 29.3 pmp in Florida. Figure 1a shows the positive correlation between AKI-D incidence and rate of renal recovery among incident ESRD patients at the level of the state (unadjusted *r* = 0.67; *p* = 0.002).

In our sensitivity analyses using data from all 25 states (44,152 AKI-D hospitalizations), we saw a similar positive correlation between AKI-D incidence and rate of renal recovery among incident ESRD patients at the state level (unadjusted *r* = 0.68; *p* < 0.001) (Fig. 1b). Similar results were seen when we used a more liberal definition of recovery (this resulted in a 1.13-fold increase in number of patients classified as recovered in the 18 states) (unadjusted *r* = 0.73, *p* = 0.001) or when we only considered cases which recovered within 6 months (unadjusted *r* = 0.61, *p* = 0.006).

In subgroup analyses, there was evidence of correlation between AKI-D incidence and rate of renal recovery among incident ESRD patients in all subgroups (Additional file 1: Figure S2, Additional file 1: Figure S3).

In partial correlation analyses, we also found a significant and positive correlation between AKI-D incidence and rate of renal recovery among incident ESRD patients after adjusting for age (partial correlation *r* = 0.62; *p* < 0.001) and sex (partial correlation *r* = 0.65; *p* < 0.001).

There was no statistically significant correlation between the number of board-certified nephrologists pmp and rate of renal recovery among incident ESRD patients (*r* = − 0.31; *p* = 0.22).

## Discussion

In this national cross-sectional ecological study conducted at the state level, we found a strong correlation between AKI-D incidence and rate of renal recovery among incident ESRD patients in 2011. These data support the hypothesis that AKI-D may be a key driver of renal recovery trends among incident patients deemed to have ESRD.

There are several reasons why AKI-D could be misdiagnosed as ESRD. Chronic kidney disease (CKD) is a strong risk factor for AKI-D and severity of CKD is a strong predictor of non-recovery after AKI-D [16, 17], making it challenging for clinicians to predict whether AKI-D patients will recover sufficiently to be able to discontinue dialysis or not.

Our findings serve to remind clinicians that some incident dialysis patients who are labeled as “ESRD” could potentially recover. Thus, it is important for providers to understand the details surrounding dialysis initiation, particularly when patients transfer care to a new outpatient nephrologist. Extra efforts need to be made to obtain medical records surrounding dialysis initiation, including knowledge of pre-AKI level of estimated glomerular filtration rate (eGFR) and amount of proteinuria which are strongly correlated with chances of kidney recovery to come off dialysis [17]. Knowing that certain patients may recover may lead to different clinical decision-making (e.g., tolerating more liberal blood pressure targets, avoiding nephrotoxins more aggressively, implementing repeated monitoring of residual renal function or urine output volume, and making more frequent nephrologist rounds). Future studies that examine optimal monitoring and treatment of AKI-D patients will be informative.

Our findings have policy-level implications as well. National health improvement targets are set using USRDS data. For example, one of the Healthy People 2020 goals is to reduce ESRD incidence by 10% [18]. Given that thousands of people with incident ESRD each year may regain enough renal function to discontinue dialysis, ESRD incidence estimates corrected for recovery might be considered as a superior metric to track disease burden. In addition, given that many AKI-D patients recover only after several months [16, 19], it may be unreasonable to expect clinicians to classify definitively whether a patient has ESRD or not as soon as he or she transitions to outpatient dialysis (via the CMS 2728 form [20] used to register patients into the USRDS ESRD database). Perhaps a “follow-up CMS 2728” form to confirm ESRD status would be beneficial to confirm ESRD for insurance and other considerations.

Further research into regional variation in rates of acute and chronic kidney disease that may shed light into pathophysiology and help identify ways to improve care. For example, studies of the impact of climate differences on AKI incidence may be fruitful [21, 22]. Other possibilities include different causes of AKI by region or the contribution of patient case-mix to recovery after AKI.

Strengths of our study include our large sample of hospitalization data from 19 states (and sensitivity analysis including 25 states) and the nationally comprehensive USRDS data. We strove for accurate capture of AKI-D cases by excluding patients with an ESRD diagnosis on admission. We also checked readmission codes of hospitalizations to ensure that it was appropriate to consider AKI-D hospitalization incidence as representing AKI-D incidence. It is also reassuring that our primary, subgroup, and sensitivity analyses yielded similar results.

Several limitations of our study should be noted. We did not have access to actual clinical data, such as serum creatinine, but relied on the best-validated set of ICD-9 diagnostic and procedure codes to define AKI-D [7, 8]. Some bias and imprecision may have been introduced into our analyses due to different SID datasets having a different number of diagnostic and procedure codes. We only counted renal recovery cases among those reported to USRDS as having ESRD. Patients with AKI-D who transitioned to outpatient dialysis units but who were not reported to USRDS were not included. Thus, the actual number of affected patients may be larger. Our analyses were limited to one calendar year (2011). We did not have SID data from all 50 states and we were unable to track readmission across multiple calendar years.

We acknowledge that this is an ecological analysis and hence subject to ecological fallacy. Our subgroup analyses by age and gender provide some reassuring data (i.e. we did not observe that all the AKI-D cases were in men and all the recovered ESRD cases were in women in a given state). We do not have individual patient level data (including accurate information regarding underlying etiology of AKI-D) and were limited in what we could adjust for on a state-level. It is possible that our results are confounded by state-level difference in practice or policy which would increase both AKI-D incidence and likelihood of recovery from ESRD within a state. However, we are not aware of any information suggesting there are important geographic variations in pertinent practice or policy by state. It is possible that practices could be inconsistent across geographic regions and could possibly have influenced our results, for example, earlier dialysis in the course of AKI in one state more than another would inflate number of AKI-D cases as well as increase the number of ESRD patients who eventually recover. The lack of correlation between the number of nephrologists and AKI-D incidence (arguing against supplier-induced demand) provides some reassurance. Furthermore, we believe our conclusion from this ecological study that AKI-D may be a key driver of renal recovery trends among incident patients deemed to have ESRD is strengthened by biological plausibility. Patients arrive at ESRD through only one of two possible disease pathways—slowly progressive CKD or AKI-D (not infrequently superimposed on CKD). The latter may recover vs. the former will not. Thus it is not surprising that states which have higher rates of AKI-D pmp also have higher rates of recovery from AKI-D pmp.

## Conclusions

Renal recovery among incident ESRD patients is not uncommon but few research publications have focused on this phenomenon and its implications for reporting and for setting public policy. To our knowledge, our study is the first to describe a geographic correlation between AKI-D incidence and renal recovery rates among patients starting maintenance dialysis at a state level. Our findings suggest that AKI-D incidence is an important driver of renal recovery rates among incident ESRD patients and raise important considerations regarding optimal care for AKI-D patients who continue to require dialysis after hospital discharge.

## Additional file

Additional file 1: **Figure S1.** Flow diagram showing selection of state for data analysis. **Figure S2.** Dialysis-requiring acute kidney injury (AKI-D) incidence vs. rate of renal recovery among incident end-stage renal disease (ESRD) patients per million population across states by sex. A) male, B) female. **Figure S3.** Dialysis-requiring acute kidney injury (AKI-D) incidence vs. rate of renal recovery among incident end-stage renal disease (ESRD) patients per million population across states by age group. A) age 45-64 years, B) age 65-74 years, C) age 75 years or older. **Table S1.** The number of dialysis-requiring acute kidney injury (AKI-D) hospitalization, AKI-D incidence, the number of renal recovery cases among incident end stage renal disease (ESRD) patients, and renal recovery rate among incident ESRD patients in 18 states. (DOCX 499 kb)

## Data Availability

The data that support the findings of this study are available from the United States Renal Data System (USRDS) but governed by a USRDS Agreement for Release of Data (DUA 2017–14).

## Abbreviations

AKI-D: Acute kidney injury requiring dialysis
AMA: The American Medical Association (AMA)
AR: Arkansas
AZ: Arizona
CA: California
CO: Colorado
eGFR: Estimated glomerular filtration rate
ESRD: End stage renal disease
FL: Florida
HCUP: The Healthcare Cost and Utilization Project
IA: Iowa
ICD-9: International Classification of Diseases Ninth Revision
KY: Kentucky
MA: Massachusetts
MD: Maryland
ME: Maine
MI: Michigan
MS: Mississippi
NC: North Carolina
NE: Nebraska
NJ: New Jersey
NM: New Mexico
NV: Nevada
NY: New York
OR: Oregon
PPD: Physician Professional Data
RI: Rhode Island
SC: South Carolina
SID: State Inpatient Databases
USRDS: The US Renal Data System
UT: Utah
VT: Vermont
WA: Washington
WV: West Virginia
