# Supplementary material for: The relation between dialysis-requiring acute kidney injury and recovery from end-stage renal disease: a national study
Source: BMC Nephrol. 2019 Sep 2;20:342. doi: 10.1186/s12882-019-1483-y (PMC6720077; doi:10.1186/s12882-019-1483-y)
Supplement: Supplementary file 1 — Figure S1. Flow diagram showing selection of state for data analysis. Figure S2. Dialysis-requiring acute kidney injury (AKI-D) incidence vs. rate of renal recovery among incident end-stage renal disease (ESRD) patients per million population across states by sex. A) male, B) female. Figure S3. Dialysis-requiring acute kidney injury (AKI-D) incidence vs. rate of renal recovery among incident end-stage renal disease (ESRD) patients per million population across states by age group. A) age 45-64 years, B) age 65-74 years, C) age 75 years or older. Table S1. The number of dialysis-requiring acute kidney injury (AKI-D) hospitalization, AKI-D incidence, the number of renal recovery cases among incident end stage renal disease (ESRD) patients, and renal recovery rate among incident ESRD patients in 18 states. (DOCX 499 kb) [file 12882_2019_1483_MOESM1_ESM.docx]

**Additional file 1**

**Figure S1:** Flow diagram showing selection of state for data analysis.

**Figure S2:** Dialysis-requiring acute kidney injury (AKI-D) incidence vs. rate of renal recovery among incident end-stage renal disease (ESRD) patients per million population across states by sex. A) male, B) female

**Figure S3:** Dialysis-requiring acute kidney injury (AKI-D) incidence vs. rate of renal recovery among incident end-stage renal disease (ESRD) patients per million population across states by age group. A) age 45-64 years, B) age 65-74 years, C) age 75 years or older.

**Table S1:** The number of dialysis-requiring acute kidney injury (AKI-D) hospitalization, AKI-D incidence, the number of renal recovery cases among incident end stage renal disease (ESRD) patients, and renal recovery rate among incident ESRD patients in 18 states

| State | Population | Number of AKI-D hospitalizations | AKI-D incidence (per million population) | Number of renal recovery cases among incident ESRD patients | Renal recovery rate among incident ESRD patients (per million population) |
| --- | --- | --- | --- | --- | --- |
| Arkansas | 2,938,640 | 982 | 334.2 | 72 | 24.5 |
| Arizona | 6,465,488 | 2143 | 331.5 | 122 | 18.9 |
| California | 37,672,654 | 9307 | 247 | 801 | 21.3 |
| Florida | 19,097,369 | 6098 | 319.3 | 559 | 29.3 |
| Iowa | 3,063,690 | 659 | 215.1 | 38 | 12.4 |
| Kentucky | 4,368,505 | 1518 | 347.5 | 98 | 22.4 |
| Massachusetts | 6,612,178 | 1472 | 222.6 | 58 | 8.8 |
| Maryland | 5,843,115 | 1633 | 279.5 | 107 | 18.3 |
| Michigan | 9,876,199 | 3179 | 321.9 | 274 | 27.7 |
| New Jersey | 8,844,694 | 2157 | 243.9 | 91 | 10.3 |
| New Mexico | 2,077,744 | 347 | 167 | 25 | 12 |
| Nevada | 2,718,170 | 1333 | 490.4 | 56 | 20.6 |
| New York | 19,526,372 | 4468 | 228.8 | 233 | 11.9 |
| Oregon | 3,865,845 | 702 | 181.6 | 44 | 11.4 |
| Rhode Island | 1,052,154 | 196 | 186.3 | 16 | 15.2 |
| South Carolina | 4,672,744 | 1071 | 229.2 | 69 | 14.8 |
| Vermont | 626,210 | 62 | 99 | 8 | 12.8 |
| Washington | 6,819,155 | 1264 | 185.4 | 75 | 11 |
